# Measuring bilayer surface energy and curvature in asymmetric droplet interface bilayers

**DOI:** 10.1098/rsif.2018.0610

**Published:** 2018-11-21

**Authors:** Nathan E. Barlow, Halim Kusumaatmaja, Ali Salehi-Reyhani, Nick Brooks, Laura M. C. Barter, Anthony J. Flemming, Oscar Ces

**Affiliations:** 1 Department of Chemistry, Imperial College London, London SW7 2AZ, UK; 2 Institute of Chemical Biology, Imperial College London, London SW7 2AZ, UK; 3 FABRICELL, Imperial College London, London SW7 2AZ, UK; 4 Department of Physics, Durham University, South Road, Durham DH1 3LE, UK; 5 Syngenta, Jealott's Hill International Research Centre, Bracknell RG42 6EY, UK

**Keywords:** droplet interface bilayers, surface energy, membrane asymmetry

## Abstract

For the past decade, droplet interface bilayers (DIBs) have had an increased prevalence in biomolecular and biophysical literature. However, much of the underlying physics of these platforms is poorly characterized. To further our understanding of these structures, lipid membrane tension on DIB membranes is measured by analysing the equilibrium shape of asymmetric DIBs. To this end, the morphology of DIBs is explored for the first time using confocal laser scanning fluorescence microscopy. The experimental results confirm that, in accordance with theory, the bilayer interface of a volume-asymmetric DIB is curved towards the smaller droplet and a lipid-asymmetric DIB is curved towards the droplet with the higher monolayer surface tension. Moreover, the DIB shape can be exploited to measure complex bilayer surface energies. In this study, the bilayer surface energy of DIBs composed of lipid mixtures of phosphatidylgylcerol (PG) and phosphatidylcholine are shown to increase linearly with PG concentrations up to 25%. The assumption that DIB bilayer area can be geometrically approximated as a spherical cap base is also tested, and it is discovered that the bilayer curvature is negligible for most practical symmetric or asymmetric DIB systems with respect to bilayer area.

## 1. Introduction

Droplet interface bilayers (DIBs) [1,2] have typically been used to measure membrane bilayer characteristics such as permeability, membrane protein interactions or electrical behaviour. Additionally, interesting DIB morphological behaviour has been studied such as bilayer area modulation by mechanical oscillation [3], membrane capacitance [4] or evaporation from the aqueous phase [5]. On a practical level, DIBs have been shown to be particularly useful as they allow for the production of asymmetric membranes [6,7], where understanding membrane asymmetry is of high value as it is known to offset transmembrane potential [8,9], affect membrane bending rigidity [10], and control membrane protein conformation [11] as well as membrane permeability [12–14].

Surface energy *γ* in bio-membranes is important to quantify as it is known to affect cellular functions such as membrane fusion, ion binding [15] and integral protein activity [16]. However, measuring surface tension in lipid DIB membranes is challenging, and, currently, the only accepted measurement method is made via direct visualization of the surface morphology using bright field microscopy, which, along with known monolayer surface tensions, can be used to infer bilayer tension. This technique, established by many groups [8,17–21], outputs a bilayer surface tension of the order of 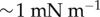 for DIBs made with lipids such as 1,2-diphytanoyl-*sn*-glycero-3-phosphatidylcholine (DPhPC). For a frame of reference, note that, according to Kwok and Evans [22], the lysis tension for lecithin vesicles was found to be of the order of 3–4 mN m^−1^. Notably, this high surface tension value (close to known rupture tensions) deviates from that of the vesicular analogue membrane tension, which is often assumed to be negligible [23]. For example, from optical techniques (laser tweezer traps), membrane tethers have been measured to have a surface tension of 3 × 10^−3^ mN m^−1^ [24]. Vesicle fluctuation analysis can also be used to estimate vesicle membrane tension of as low as 10^−3^ mN m^−1^ [25]. The surface tension of neutrophils has been calculated to be 0.03 mN m^−1^ [26], measured with micropipette aspiration [27,28] or the micropipette interfacial area-expansion method [29]. The lipid 1,2-dioleoyl-*sn*-glycero-3-phospho-(1′-rac-glycerol) (DOPG) was chosen as it is documented that the uncharged PC lipids reduce the surface tension of pulmonary surfactants that contain a large amount of the charged PG lipid [30,31].

Certainly, as DIB membranes are high-energy systems relative to their vesicular counterparts, measuring membrane tension in DIBs is unfortunately limited by stability. Furthermore, as DIB membrane oscillation cannot be captured optically, and as micropipette aspiration of DIBs would not affect any change in surface tension, it appears that morphological measurements are the only practical option to calculating surface tension. However, though it has been shown that symmetric DIB bilayer surface energies can be estimated using shape information from bright field images, bright field microscopy lacks the ability to capture precise information about membrane curvature due to lipid asymmetry, which can significantly affect the surface energy calculation.

In this study, for the first time using confocal laser scanning fluorescence microscopy (CLSM), it is shown that, in DIBs composed of droplets of different volumes, there exists curvature in asymmetric bilayers of lipids with differing surface properties. CLSM was required as it provided a higher resolution image for the bilayer shape which is not obscured by extraneous light from above and below the midplane of the DIB. This shape information can be applied to the calculation of membrane tension in accordance with a force balance, i.e. Neumann's triangle [32] (the sine rule). Additionally, a free energy model is applied that describes the curvature behaviour with respect to lipid asymmetry and droplet volume difference.

## 2. Material and procedures

### 2.1. Lipid preparation

The lipids DPhPC, DOPG and 1-oleoyl-2-[12-[(7-nitro-2-1,3-benzoxadiazol-4-yl)amino]dodecanoyl]-*sn*-glycero-3-phosphocholine (NDB-PC) were purchased from Avanti Polar Lipids. Samples were prepared with 10 mg of solid lipid mixtures suspended in chloroform. The suspension was evaporated to give a film deposited on the vial surface. The film was desiccated for 30 min and re-suspended in a 0.25 M phosphate buffer solution at pH 7.4. The samples underwent freeze–thaw cycles in liquid nitrogen and in a water bath at 60°C, repeated five times each. The frozen samples were stored at −20°C until used. Before use, the samples were thawed and diluted to 5 mg ml^−1^ and extruded 11 times through 100 nm Avanti PC membrane filters. For CLSM, the fluorescent lipid NBD-PC was similarly deposited on a vial surface and was suspended in the previously extruded lipid solutions to a molar concentration of 0.1%. It was assumed that the low concentration of NBD-PC does not appreciably affect the surface properties of the lipid monolayer or bilayer.

### 2.2. DIB formation

DIBs were formed by pipetting lipid-in aqueous emulsions into acrylic wells filled with hexadecane. Acrylic wells are chosen for DIB manifolds as the droplet wettability was reduced and has a refractive index of 1.49 [33], which was not dissimilar to the supplier reported value for hexadecane at 1.43. DIBs were formed at 5 mg ml^−1^ lipid concentration. The dynamics of monolayer formation have already been established [34], which show that lipid-in DIBs require a short incubation period of the order of minutes as single droplets in hexadecane before they are pushed together with a needle to form interfaces. There is also a period of the order of minutes where the DIBs ‘zip up’ to form a bilayer at equilibrium; curvature measurements are taken at this equilibrium state. Note it is assumed that negligible amounts of residual oil may be trapped in the bilayer, as previous experiments have shown that this DIB system can accommodate the mechanosensitive membrane protein MscL and retain functionality [35].

### 2.3. Confocal microscopy

A Leica TCS SP5 confocal fluorescent microscope was used with a 10× objective set with an 84.5 µm pinhole (1 airy unit). The field of view was set to 775 × 775 µm (512 × 512 pixels) and the samples were acquired at a frequency of 400 Hz with eight line averages. The excitation was achieved with three wavelengths of 458, 476 and 488 nm and absorbance was set at between 510 and 550 nm. The images used to fit the model were acquired in the midplane of the droplets. During data collection, focal planes slightly above and below were viewed to confirm that the image was indeed acquired from the midplane.

### 2.4. Pendant drop measurements and drop shape analysis

It has been shown by Lee *et al.* [36] that ionic screening of PC/PG vesicles is required to allow the lipids to coat an air/water monolayer surface. In order to confirm that the lipids have absorbed on the monolayer drop shape analysis (DSA) measurements can also be performed on the lipid solutions in hexadecane. Surface energies of mixtures of lipids were calculated with a pendant tensiometer (Krüss) by DSA. The lipids used for making DIBs were formed into aqueous droplets and were immersed in hexadecane from a flat needle 0.52 mm in diameter. The Worthington number *Wo* [37]2.1
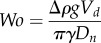
is a dimensionless number which measures the ratio of gravitational to surface forces and is an analogue of the well-known Bond number 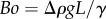 in bubble systems, where *L* is the characteristic length [38]. It is often used to estimate the accuracy of the DSA technique, where a measurement is considered accurate at around *Wo* ∼ 1 and inaccurate for *Wo* ≪ 1 [39]. Thus, the volume of the droplet must be maximized in order to attain accurate surface energy measurements. For this system, the density difference between water and hexadecane is Δ*ρ* = 230 kg m^−3^, acceleration of gravity *g* = 9.8 m s^−2^, droplet volume *V_d_* = 0.1 − 0.5 ×10^−9^ m^3^, needle diameter *D_n_* = 5.2 × 10^−4^ m and surface tension *γ* is of the order of 10^−3^ J m^−2^ [39]. Owing to the low adhesion energy of the DPhPC and DOPG monolayers, pendant drop measurements become troublesome as the gravitational potential energy of large droplets overwhelms the pendant droplet adhesion and falls off the needle before equilibrium is reached. This limits the possible range of experimental values of the *Wo* to between 0.26 and 0.99.

## 3. Results and discussion

### 3.1. Model equation and geometry

**Figure 1.**
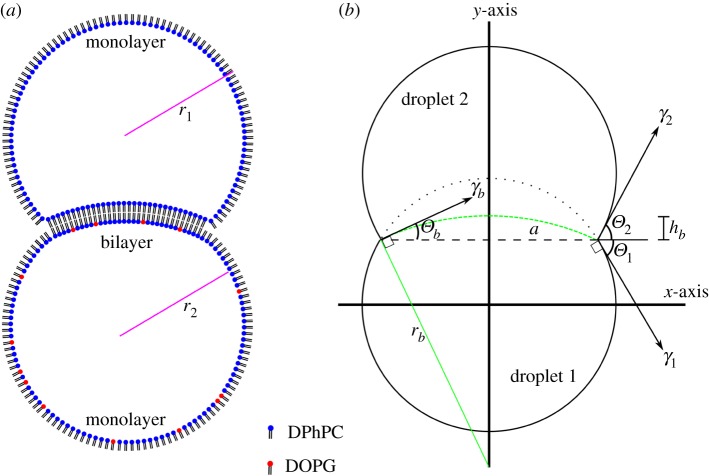
(*a*) Diagram depicting an asymmetric DIB with one droplet of radius *r*_1_ formed from DPhPC vesicles and one droplet formed from DPhPC doped with DOPG lipids of radius *r*_2_. (*b*) Diagram of an asymmetric DIB which exhibits curvature in the bilayer with surface energy *γ_b_* of radius *r_b_* and with angle relative to the *x*-axis, *Θ_b_*, which balances the surface energies *γ*_1_ and *γ*_2_ with contact angles *Θ*_1_ and *Θ*_2_. *h_b_* is the spherical cap height of the bilayer.

It is shown that there may exist a bend in the bilayer between the droplets that form a DIB [18]. Under the assumption that the DIB retains axial symmetry, as demonstrated in figure 1, the bilayer bend of radius *r_b_* can be modelled as a section of a spherical cap of height *h_b_* and the droplets themselves can be modelled as intersecting spheres of radius *r*_1_ and *r*_2_ truncated at height *h*_1_ and *h*_2_ with spherical cap base radius *a*. Note that there is an important distinction between the effective bilayer curvature (1/*r_b_*) in a DIB and the lipid spontaneous curvature *c*_0_ [40]. The curvature in the DIB is a non-local description of the droplet macrostructure. In this work, the lipids DOPG and DPhPC are used as they form stable bilayers [41] with differing surface energies. Though both DPhPC [42] and DOPG [43] have negative spontaneous curvature, planar and positive curvature can occur in DIB membranes.

By setting the bilayer concavity towards droplet 2 (figure 1), owing to a surface tension force balance [32] the equations3.1
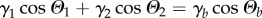
and3.2
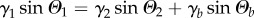
must hold for a given set of bilayer and droplet monolayer surface energies *γ_b_*, *γ*_1_ and *γ*_2_. This is equivalent to the analysis carried out by several authors [8,17–21]. The bilayer and droplet contact angles *Θ_i_* (where the index *i* is the set [*b*, 1, 2]) are defined in figure 1*b*.

In practice, as droplet radius and position are relatively easy to measure and can be used to measure contact angles *Θ*_1_, *Θ*_2_ and *Θ*, the force balance of (3.1) and (3.2) can expressed as3.3
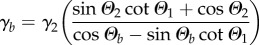
and3.4
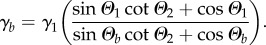


The usefulness of the form in (3.3) and (3.4) becomes apparent if the DIB geometry is known along with single surface energy value *γ*_1_ or *γ*_2_, in which case the bilayer surface energy *γ_b_* can then be calculated. Thus, based on this a single experimental value of surface energy, consisting of both bilayer and monolayer surface energies, can be calculated using geometric information from CLSM DIB images. The error propagation analysis of this equation is provided in the electronic supplementary material.

In §3.5, we will theoretically consider how DIB asymmetry affects the bilayer area and curvature. To write down a set of equations which are uniquely solvable, we will assume that the volumes of the droplets are known and conserved. Using simple geometry, 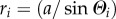, and the standard equation for the volume of a spherical cap, the volumes of droplets 1 and 2 are3.5
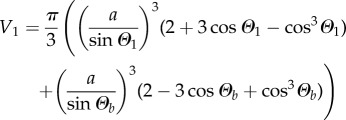
and3.6
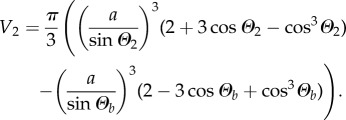
Now that four equations and four variables remain—namely equations (3.1), (3.2), (3.5) and (3.6) with variables *Θ*_1_, *Θ*_2_, *Θ_b_* and *a*—the system of equations can be solved. However, as there is no simple analytic solution, this system must be solved using numerical techniques.

### 3.2. Symmetric lipid DIB confocal imaging result

**Figure 2.**
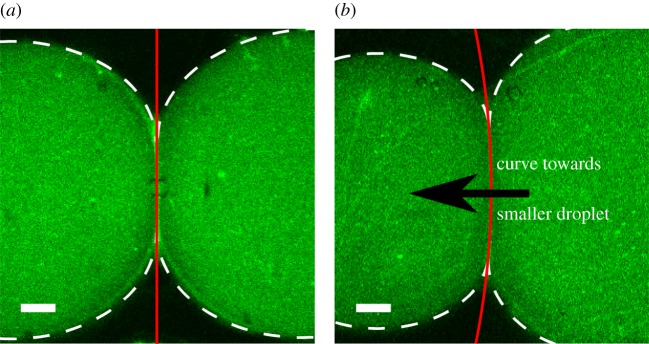
Filtered CLSM image (*a*) of a volume-symmetric DIB formed from a single lipid type and (*b*) of a volume-asymmetric DIB (volume ratio of 0.37) that exhibits bilayer interface curvature. Both droplets consist of DPhPC with dilute (0.1% molar fraction) NDB-PC dye formed in hexadecane. The bilayer concavity faces the smaller droplet with higher Laplace pressure. Scale bars, 100 µm.

As an experimental control, symmetric lipid DIBs were formed as shown in figure 2. Here, the monolayer surface energy of a pure DPhPC monolayer between water and hexadecane is taken as 1.18 mN m^−1^ [17,34]. A DIB made up of pure DPhPC with closely matching volumes that vary by less than 1% is shown under CLSM to exhibit no bilayer curvature. To verify that there is no appreciable bilayer curvature, the image is processed with standard techniques using the Matlab image processing toolbox. All the original data are processed with a Gaussian filter to smooth the edges on the interface peaks and the Matlab ‘fminsearch’ function was used to attempt to fit the interface shape to the equation of a circle and to a line. Unsurprisingly, the solver could not fit the interface to the equation of a circle, but could fit to a straight line with a root mean square error (RMSE) of 0.15, depicted as a red line in figure 2*a*. The droplet positions and radii are measured using the Matlab function ‘imfindcircles’, which employs the Hough [44] transform. The dimensions of the symmetric DIB in figure 2*a* were found to be *r*_1_ = 433 µm, *r*_2_ = 437 µm, *r_b_* = inf, and *a* = 221 µm. From equations (3.3) and (3.4), with an input value of *γ*_1_ = *γ*_2_ = 1.18 mN m^−1^, the bilayer surface energy was calculated to be *γ_b_* = 2.04 mN m^−1^ with an error of 0.121 mN m^−1^ (see the electronic supplementary material for error propagation analysis), matching previously reported surface energy results from Taylor *et al.* [17].

By contrast, a non-similar volume DIB (i.e. a volume ratio of 0.37) is shown to exhibit a circular curve in the bilayer which bends toward the smaller droplet, shown in figure 2*b*. To calculate the bilayer curvature, the image is processed again in Matlab using image processing. Within the region of interest, the maximum intensity peak values are obtained along the vertical axis. These peak values are fitted to the equation of a circle using the Matlab function ‘fminsearch’ to minimize the RMSE of the distance from a peak point to the fitted circle. The droplet dimensions are also measured using the Matlab function ‘imfindcircles’. From this, the ratio of the bilayer radius of curvature to the smaller droplet radius of curvature in the figure is measured to be 7.21 with an RMSE of 0.12, depicted as a red line. Based on the measured, normalized geometry of *r*_1_ = 397 µm, *r*_2_ = 535 µm, *r_b_* = 2859 µm and *a* = 270 µm, the surface energy for the bilayer is calculated to be *γ_b_* = 1.93 mN m^−1^ with an error of 0.107 mN m^−1^. Here the actual bilayer surface energy measurement is within the error of the previously reported measurement, 2.04 mN m^−1^ [17].

### 3.3. Asymmetric lipid DIB confocal imaging result

**Figure 3.**
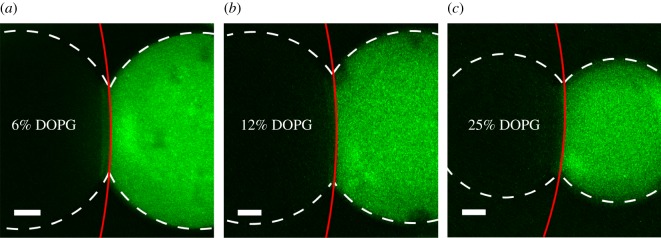
The filtered CLSM images of matching volume droplet DIBs in hexadecane with mismatched surface energies exhibit curvature in the bilayer towards the higher surface tension droplet. The dark droplet contains the DOPG and light droplet contains the DPhPC with NDB-PC. The amount of DOPG in (*a*), (*b*) and (*c*) is, respectively, 6%, 12% and 25% from total lipid content. Scale bars, 100 µm.

The results of the CLSM experiment on asymmetric DIBs are provided in figure 3. Three DIBs of varying degrees of monolayer asymmetry, from lowest to highest, are shown to exhibit bilayer curvature, where the interface curvature is measured with a Matlab script in which the derivative of the fluorescence intensity plot is used to find the image edge threshold, which is fitted to the equation of a circle by minimizing the RMSE; see the electronic supplementary material for more details. The geometry furthermore can be used to calculate the bilayer and the monolayer surface energy. Note that in the following cases the pure DPhPC lipid droplet surface energy is assumed to remain at *γ*_2_ = 1.18 mN m^−1^. Figure 3*a* shows a bilayer curvature to droplet curvature ratio of 4.96 and a spherical cap base radius to droplet radius ratio of 0.44 at an RMSE of 0.31. Note that in figure 3*a* the monolayer in the dark (leftmost) droplet 1 is composed of 6% DOPG from total lipid content, which is left dark to enhance the contrast in the bilayer threshold. The geometric measurements of the DIB are *r*_1_ = 431 µm, *r*_2_ = 431 µm, *r_b_* = 2138 µm and *a* = 194 µm, where a brightfield image of the dark droplet is used to measure the dimensions of the dark droplet. The increased surface energy for droplet 1 and the bilayer is calculated to be *γ*_1_ = 1.70 mN m^−1^, and *γ_b_* = 2.58 mN m^−1^ with an error of 0.149 mN m^−1^.

Further increasing the DIB asymmetry shown in figure 3*b* confirms that the bilayer radius of curvature ratio deceases to 3.34 with a spherical cap base radius to droplet radius ratio of 0.49 at an RMSE of 0.33. The asymmetric DIB is composed of 12% DOPG from total lipid content in the left droplet with dimensions measured to be slightly volume asymmetric, *r*_1_ = 452 µm, *r*_2_ = 428 µm, *r_b_* = 1420 µm and *a* = 225 µm. Similarly, the surface energies for droplet 1 and the bilayer are calculated to be *γ*_1_ = 1.92 mN m^−1^ and *γ_b_* = 2.68 mN m^−1^, respectively, with an error of 0.169 mN m^−1^.

The third and highest stable asymmetric DIB formed in figure 3*c* is composed of 25% DOPG from total lipid content. The bilayer radius of curvature ratio is measured at 2.23 and spherical cap base radius to droplet radius ratio of 0.58 with an RMSE of 0.516. The DIB dimensions are calculated to be *r*_1_ = 334 µm, *r*_2_ = 331 µm, *r_b_* = 738 µm, and *a* = 185 µm, where the surface energy for droplet 1 and the bilayer is calculated to be *γ*_1_ = 2.70 mN m^−1^ and *γ_b_* = 3.33 mN m^−1^ with an error of 0.195 mN m^−1^.

### 3.4. Pendant drop measurements (DSA) results

To compare with the above DIB method of measuring surface energy, the results from the DSA measurements are provided in table 1. The measurement was taken for droplets that could attain equilibrium without falling from the flat syringe needle. Note that the Worthington number (*Wo*) is close to 1 for most measurements. The results below can be used to verify the DIB morphology method for surface tension.

**Table 1.** Table of surface energy measurements for a droplet of DPhPC with a given percentage of DOPG that forms a monolayer between water and hexadecane. The error and Worthington number are provided for reference.

per cent DOPG	*γ*_1_, mN m^−1^	s.d.	*Wo*
50.0	3.13	1.56	0.26
25.0	2.65	1.35	0.71
12.5	2.02	1.02	0.67
6.3	1.74	0.91	0.99
0.0	1.50^a^	0.82	0.70

^a^Note that the literature value for pure DPhPC is 1.18 mN m^−1^ [17,34].

**Figure 4.**
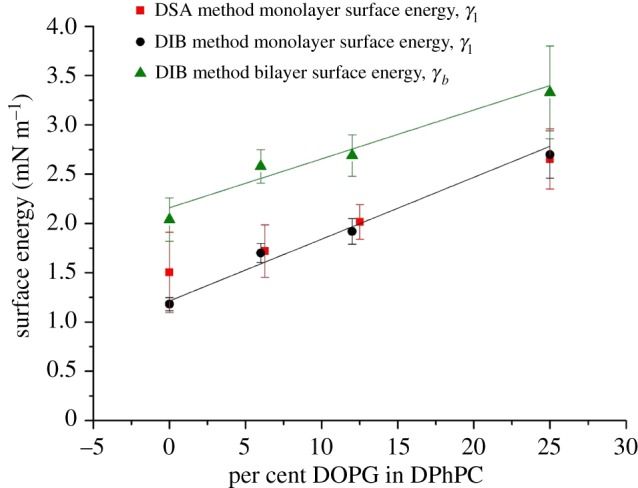
Bilayer and monolayer surface energies obtained from DSA and DIB methods as a function of monolayer asymmetry in DIBs with a droplet composed of pure DPhPC and a droplet with a mixture of DOPG in DPhPC. For the DIB method, the droplet 2 surface energy is assumed to be *γ*_2_ = 1.18 mN m^−1^ for pure DPhPC. The linear fits for the bilayer and monolayer surface energies have a Pearson's *R*^2^-value of 0.94 and 0.98, respectively, with a sample size of *n* = 3.

The DSA results show good agreement with the DIB method, as shown in figure 4. This verifies the technique developed by several authors with respect to symmetric DIBs [8,17–21]. However, here we have shown that, with the use of CLSM, we can capture bilayer curvature data to be used in calculating asymmetric bilayer surface energy. This is useful as with significantly low surface energies (lower than 5 mN m^−1^) it is often difficult to obtain shape measurements from the standard DSA method as the droplets tend to fall from the needle [34]. Given that they are stable and stationary, by the DIB method, asymmetric bilayer surface energies can be calculated. We further note that the discrepancy in the measurement of pure DPhPC comes chiefly from the fact that DSA measurements become more difficult with low surface tensions, in this case *γ* < 1.5 mN m^−1^.

### 3.5. Droplet morphology model result

**Figure 5.**
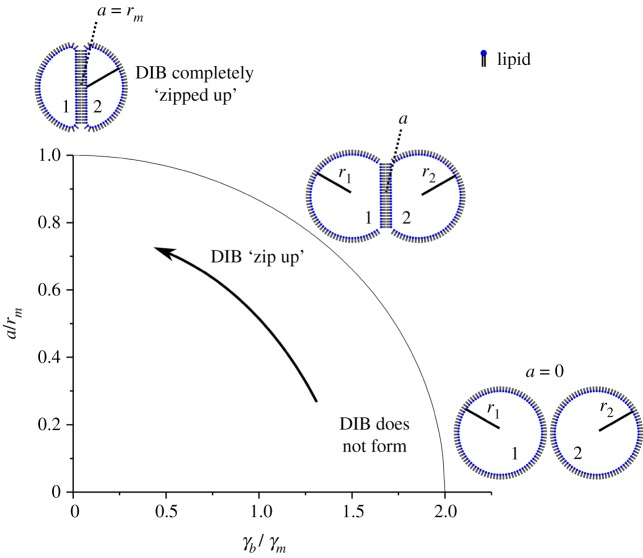
Symmetric DIB model result for the ratio between the spherical cap base radius *a* and the droplet radii *r*_1_ and *r*_2_ as a function of bilayer to monolayer tension *γ_b_*/*γ_m_*. The droplets ‘zip up’ drastically with increasing monolayer surface energy up to the point the bilayer and monolayer energies match, after which the effect is less dramatic until the droplets ‘zip up’ completely and the droplet radius matches that of the bilayer radius. Note that the lipids in the DIB diagrams are for shape reference and not drawn to scale.

The free energy model described in §3.1 was applied to investigate asymmetric and symmetric DIB morphology with the given system surface energies *γ*_1_, *γ*_2_ and *γ_b_* by equations (3.1), (3.2), (3.5) and (3.6). A simple way to analyse the system is to view the interface diameter *a* normalized by the droplet radius *r_m_*. This is useful as it can be generalized and scaled for different droplet systems driven by surface energy minimization. By this assessment, the symmetric model is 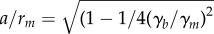, which is shown by figure 5, where the DIB monolayer surface energies *γ*_1_ and *γ*_2_ of droplets 1 and 2 are the reference values, i.e. *γ_b_* is in the form of *γ_b_*/*γ_m_*. As the bilayer surface energy is decreased from *γ_b_*/*γ_m_* = 2 or *a*/*r_m_* = 0 the DIB will start to ‘zip up’. This ‘zip up’ process is defined as an increase in contact surface area between droplets. Here one can see that the ratio of spherical cap base radius *a* to droplet radius *r*_1_ and *r*_2_ increases drastically following the arrow and gradually decreases until *γ_b_*/*γ_m_* = 0 or *a*/*r_m_* = 1. This is an unsurprising result as it has already been shown by (3.1) that the contact angle *Θ_m_* changes as 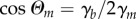. This ‘zip up’ process occurs mainly up to the point where the bilayer surface energy matches that of the monolayers, or 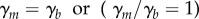. After this point, any small perturbation in bilayer surface energy will have a diminished effect on bilayer radius *a*. Note that, as surface energy is finite, the DIB can only ‘zip up’ completely if *γ_b_* = 0.

**Figure 6.**
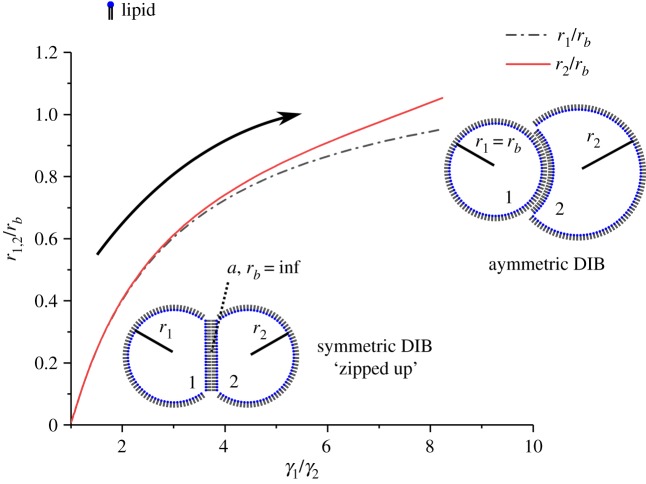
Asymmetric DIB model result for the ratio of the droplet radii *r*_1_ and *r*_2_ to bilayer radius *r_b_* as a function of monolayer tension ratio *γ*_1_/*γ*_2_. Here *γ*_2_ = *γ*_*b*_. Owing to the force balance, the bilayer deviates from the spherical cap base radius *a*, and following the arrow it will continue to curve towards the higher tension droplet until the bilayer radius matches that of the droplet radius, *r*_1_ = *r*_*b*_. Owing to a mass balance, the diameter of droplet 2 will increase above that of droplet 1. Note that the lipids are not drawn to scale in the DIB diagram.

There is no simple analytical solution to the asymmetric case. However, it can be solved using numerical techniques. A Matlab script was employed to solve for the variables *r*_1_, *r*_2_, *r_b_* and *a*. The script employs the ‘fmincon’ function, which runs an ‘interior-point’ algorithm, to solve for the minimization of the free energy functional [21] *f* of surface energy *γ* and surface area *A*,3.7
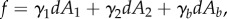
under the constraint that *V*_1_ and *V*_2_ (from equations (3.7) and (3.8)) are constant. This script was used to solve for the ratio of the drop radii *r*_1_, *r*_2_ and the bilayer radius *r_b_*. Figure 6 shows that, for asymmetric DIBs, the membrane radius *r_b_* will decrease with increasing asymmetry in monolayer surface tension *γ*_1_/*γ*_2_ until it matches the spherical cap base radius, or *r_b_* = *a*. For simplicity, here we have used *γ*_2_ = *γ_b_* as the reference tension value. Note that a DIB with a bilayer of infinite radius *r_b_* → ∞ (zero mean curvature) exists at the symmetric limit. The model result also shows that, for asymmetric DIBs greater than the range of *γ*_1_/*γ*_2_ ∼ 1.2, small changes in asymmetry affect the bilayer radius of curvature significantly.

Often bilayer area is approximated by the spherical cap base radius (or the linear distance between the intersecting circles) *A*_approx_ = *πa*^2^. The definition of bilayer area considering curvature (spherical cap area) is given as *A_b_* = 2*πr_b_h_b_*. Therefore, the per cent area deviation from the linear approximation can be defined as Δ*A*,3.8
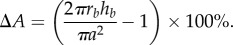


**Figure 7.**
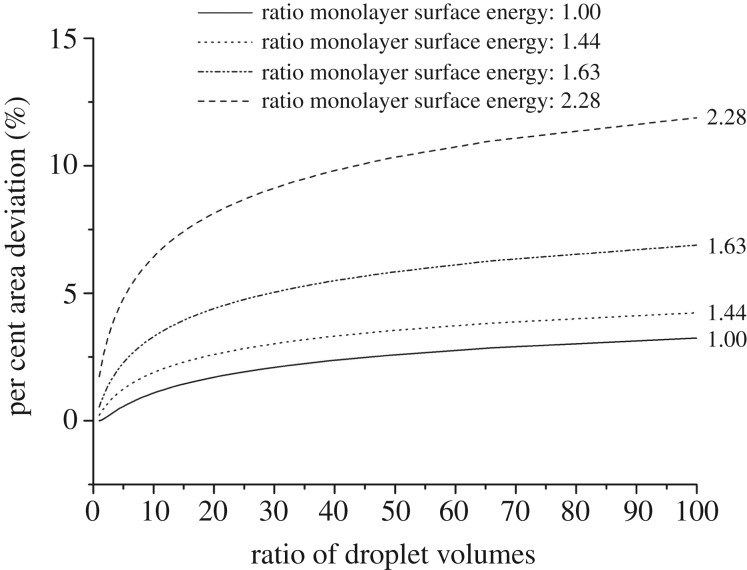
Model result of the per cent area deviation of DIBs with volume and lipid asymmetry given that the bilayer surface tension is set to the average of the two monolayer surface tensions. The effect of monolayer surface energy is exacerbated by increasing droplet volume differences.

As shown in figure 7 for volume-symmetric DIBs, increasing the monolayer asymmetry elicits only a modest deviation in surface area. However, if the droplet volume asymmetry is modified the area deviation can be magnified. Note that typically DIB droplets are roughly the same size, and high surface energy asymmetry does not appear to be stable experimentally. By applying this model, the area correction of the DIB bilayers found experimentally via CLSM can be determined. For the volume-asymmetric droplet (figure 2*b*), by equation (3.10), the area deviation Δ*A* is found to be 0.22%. Additionally, the area deviation for the lipid asymmetric DIBs is found to be 0.215, 0.635 and 1.6% for the 65, 125 and 25% DOPG in DPhPC, respectively. This shows that, at least for the range of DIB asymmetry explored in this study, the linear approximation of area is a reasonable estimate. Indeed, according to figure 7, even relatively high monolayer asymmetry manifests as a deviation of less than 5% for volume-symmetric DIBs.

### 3.6. Model and system limitations

#### 3.6.1. Practical limitations of the method

The valid range of *γ*_1_ and *γ*_2_ for the model is limited by DIB stability as experimentally stable DIBs are only formed below a surface energy ratio of 2.5. Above this level, the droplets are disposed to coalesce into one larger droplet. This can be explained by the fact that emulsion or DIB stability depends on (i) the osmotic and Laplace pressure of the droplets and (ii) the pressure balance across the membrane [45]. Inescapably, the difference in pressure between connecting droplets may lead to inherent instability for large droplet volume ratios, which limits the practicality for extreme droplet volume ratios.

#### 3.6.2. Limitations in scalability

The use of DIBs for measuring surface tension is limited in size to microscale droplets. This is the case as other thermodynamic factors come into play on smaller length scales such as line tension, which becomes non-negligible once the droplet reaches length scales below 100 nm [46]. Additionally, it is important to note that attaining thermodynamic equilibrium can be somewhat troublesome for DIBs as they continually lose water mass due to evaporation; this is a particular problem for DIBs with diameters on a micron length scale [5]. The effect of evaporation on DIBs was characterized recently by Venkatesan *et al.* [47], and this effect is mitigated by using droplets of the order of 300 µm in diameter covered by a thick layer of oil; however, the effect of gravity on droplet shape prevents the use of much larger DIBs without adding another level of complexity to the model [48]. Indeed, the model system is limited in scalability by the Bond number (see §2.4). For this study, the density difference between water and hexadecane is Δ*ρ* = 230 kg m^−3^, acceleration of gravity *g* = 9.8 m s^−2^, and surface tension *γ* is of the order of 10^−3^ J m^−2^. If the characteristic length is taken as droplet radius, then *L* is of the order of 2.3 − 4.0 × 10^−4^ m, this implies a Bond number of approximately 0.1–0.4. A reasonable upper limit for DIB applications is a Bond number less than 1 (a droplet radius of 660 µm for the system at hand), as values greater than 1 imply a decreased effect of surface tension relative to gravity and will result in non-spherical droplets. Note that this model does not account for non-spherical droplets.

#### 3.6.3. Model limitations

The model is limited to static equilibria and cannot be used to probe the absolute surface tension of the DIB membrane out of equilibrium, though the relative surface forces, such as 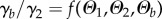, can be calculated from equations (3.3) and (3.4). In a similar vein, the model is only valid for systems that are under tension. More specifically, this model would not be particularly useful to measure the tension of adhering vesicles, as the mechanical tension is not necessarily known as the bodies can be deflated and the energetics can be affected by the expansion modulus [49].

## 4. Conclusion

For the first time, it has been shown that asymmetric DIBs form a curved surface in the bilayer due to a surface energy balance. This is analogous to the effect of volume differences, but here the surface energy asymmetry controls this behaviour. As shown by Taylor *et al.* [17] for symmetric DIBs, our study shows that the curvature effect in asymmetric DIBs can be employed as an alternative method of measuring interfacial tension of complex, asymmetric lipid monolayers or bilayers through the application of CLSM. The obtained interfacial tension values are in good agreement with droplet shape analysis results. Furthermore, the results obviate the negligible effect of area deviation with respect to DIB asymmetry; though the effect of curvature strongly affects the surface tension calculation, even the most asymmetric system in this experiment (with a surface energy ratio of approx. 2.2) corresponds to a deviation of only 1.6%. Thus, with DIB platforms, the bilayer interfacial area measurement is only affected by the extreme cases of high surface tension asymmetry and extreme volume mismatch, an important validation of an assumption made in many published DIB applications.

A linear relationship between bilayer surface energy with respect to DOPG and DPhPC mixtures is shown up to 25% DOPG. However, this linear relationship is not necessarily the case for all lipid mixtures. For example, significant nonlinearity and hysteresis in dynamic interfacial tension measurements as a function of the mole fraction of cholesterol in lecithin lipids has been observed [50]. The formation of a lipid–lipid complex has been shown for phosphatidylcholine–phosphatidylethanolamine and sphingomyelin–ceremide mixtures; this implies a nonlinear relationship for interfacial tension with respect to lipid concentrations [51]. Thus, the asymmetric DIB morphology method could be used to probe this nonlinear surface tension behaviour by measuring the surface morphology as a function of lipid content and asymmetry.

There is a wide range of possibilities for future work measuring surface tension and curvature effects in DIBs, giant unilameller vesicles [52] or even cells. It has already been shown that curvature exists between adhering cells as observed in the biologically mediated cell–cell contact between *Caenorhabditis elegans* embryos [53] and between adhering vesicles [42]. Investigations of lipid flip-flop in bio-membranes [54], to a marginal degree of success, have been performed using sum frequency vibrational spectroscopy [55], indirectly with ceremide-induced trans-bilayer movement in vesicles [54], small-angle neutron scattering [56] and by molecular simulation [57]. Asymmetric DIBs or adhering vesicles offer an alternative measurement technique for the rate of lipid flip-flop, by directly measuring the decrease of interfacial curvature as the lipids flip from one droplet or vesicle to another. Here the challenge lies in distinguishing the rate of flip-flop from the rate of lateral lipid diffusion [58] between the monolayer and bilayer, as well as lipid uptake into the bilayer [5,50,59]. However, a recent publication has demonstrated a promising technique for determining bilayer flip-flop on DIB membranes via parallel capacitance-based measurements on an integrated microfluidic device; in this study, it was successfully shown that surface-bound peptides (alamethicin) facilitate the movement of lipids between leaflets [9].

The present model is valid for stationary surfaces at equilibrium. It would also be interesting to extend the model to dynamic behaviour of micro-DIBs where the droplets change shape and the bilayer may even buckle [60]. The bilayer buckling indicates that the effective bilayer surface tension *γ_b_* had dropped to zero [5]. Understanding interfacial physical chemistry is paramount to the development of DIBs as a tool for biological discovery, which is crucial for burgeoning fields such as synthetic biology and biotechnology.

## Supplementary Material

Supplementary Information

## Data accessibility

This article has no additional data.
