# Supplementary material for: Measuring bilayer surface energy and curvature in asymmetric droplet interface bilayers
Source: J R Soc Interface. 2018 Nov 21;15(148):20180610. doi: 10.1098/rsif.2018.0610 (PMC6283991; doi:10.1098/rsif.2018.0610)
Supplement: Supplementary Information [file rsif20180610supp1.docx]

**ELECTRONIC SUPPLIMENTARY INFORMATION**

**Measuring Bilayer Surface Energy and Curvature in Asymmetric Droplet Interface Bilayers**

Nathan E. Barlow,^a,b^ Halim Kusumaatmaja,^c^ Ali Salehi-Reyhani,^a,b,e^ Nick Brooks,^a,b^ Laura M. C. Barter,^a,b^ Anthony J. Flemming,^d^ Oscar Ces^a,b,e,*^

^a^ Department of Chemistry, Imperial College London, Exhibition Road, London, SW7 2AZ, UK. *[o.ces@imperial.ac.uk](mailto:o.ces@imperial.ac.uk)

^b^ Institute of Chemical Biology, Imperial College London, Exhibition Road, London, SW7 2AZ, UK

^c^ Department of Physics, University of Durham, South Road, DH1 3LE, UK

^d^ Syngenta, Jealott's Hill International Research Centre, Bracknell, Berkshire, RG42 6EY, UK

^e^ FABRICELL, Imperial College London, London SW7 2AZ

**Image Edge Threshold**

The membrane surface area of the DIB can be extracted from image processing of the surface features of the fluorescence image. This is achieved by returning the fluorescence intensity peaks at $(\hat{x}_{i},\hat{y}_{i})$ at each row index $i$ in the region of interest as shown in Figure S.1. It is assumed a priori that the bilayer has spherical curvature and can be fit to the equation of a circle

| $r^{2}=\left( x-h \right)^{2}+\left( y-k \right)^{2}$ | (S.0) |
| --- | --- |

The bilayer radius of curvature $r$, and circle centre ($h,k$) are found by a root mean square error minimization $RMSE$ on (S.0) by varying $r,h,$and$k$ from the equation

| $RMSE=\sqrt{\frac{{\sum_{x=x_{1}}^{x_{N}} \left( \hat{y}_{i}-y_{i} \right)}^{2}}{N}}$ | (S.1) |
| --- | --- |

**
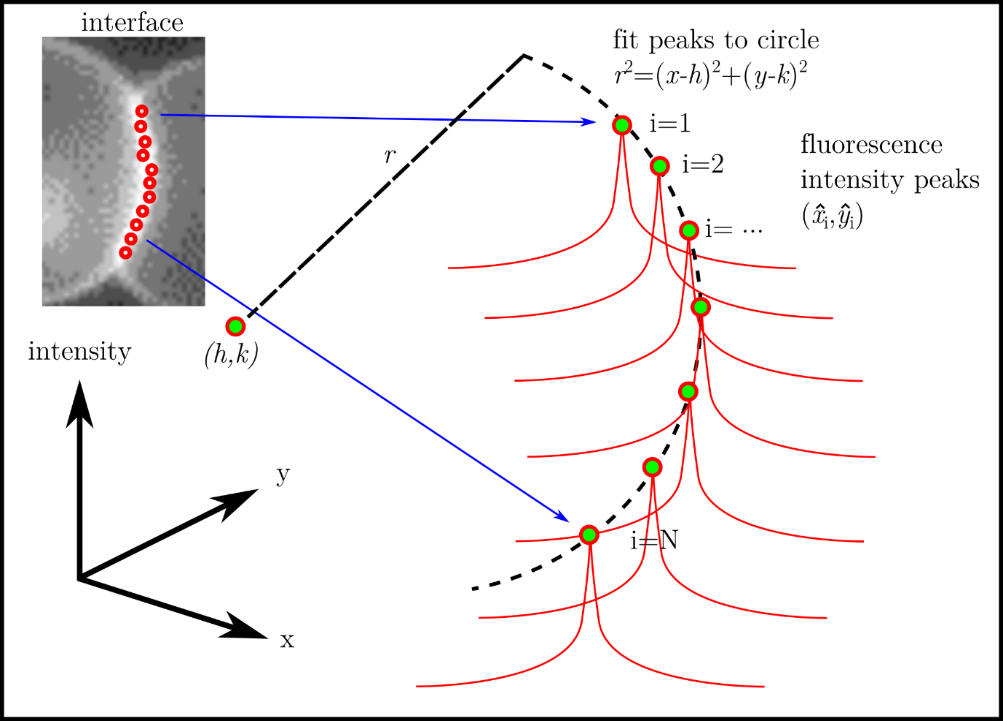
**

Figure S.1: *Diagram of feature extraction of bilayer interface, where the maximum pixel intensity value is found for each row index* $i$ *in the x-y plane. These points are then fit to the equation of a circle to output the bilayer radius* $r$ *by root mean square error minimization.*

**Free Energy Model Lagrange Multipliers Solution**

A surface energy model can be applied to the system that is equivalent to a force balance. The DIB interfacial areas and volumes can be measured to account for the bilayer curvature. Given the geometry of **Figure 3.1**, the surface area of the first droplet

| $A_{1}=4\pi r_{1}^{2}-2\pi r_{1}h_{1}$ | (S.1) |
| --- | --- |

is given by the surface of the sphere minus the spherical cap. The opposing droplet area

| $A_{2}=4\pi r_{2}^{2}-2\pi r_{2}h_{2}$ | (S.2) |
| --- | --- |

is given in a similar fashion. Finally, the bilayer surface area

| $A_{b}=2\pi r_{b}h_{b}$ | (S.3) |
| --- | --- |

is also assumed to be a spherical cap. The volumes

| $V_{1}=\frac{4}{3}\pi r_{1}^{3}-\frac{1}{3}\pi h_{1}^{2}\left( 3r_{1}-h_{1} \right)+\frac{1}{3}\pi h_{b}^{2}\left( 3r_{b}-h_{b} \right)$ | (S.4) |
| --- | --- |
| $V_{2}=\frac{4}{3}\pi r_{2}^{3}-\frac{1}{3}\pi h_{2}^{2}\left( 3r_{2}-h_{2} \right)-\frac{1}{3}\pi h_{b}^{2}\left( 3r_{b}-h_{b} \right)$ | (S.5) |

are given by the volume of sphere minus the volume of the respective spherical caps and plus or minus the bilayer spherical cap depending on which direction the bilayer curves. Note the sign difference in the opposing droplet from equation (3.10) to (3.11). By the Pythagorean Theorem, the volume and area are all related by equations

| $h_{1}=r_{1}-\sqrt{r_{1}^{2}-a^{2}}$ | (S.6) |
| --- | --- |
| $h_{2}=r_{2}-\sqrt{r_{2}^{2}-a^{2}}$ | (S.7) |
| $h_{b}=r_{b}-\sqrt{r_{b}^{2}-a^{2}}.$ | (S.8) |

Assuming that any other thermodynamic effect is negligible, the energy functional of the DIB system can be expressed as the equation

| $f=\gamma_{1}2\pi\left( r_{1}^{2}+r_{1}\sqrt{r_{1}^{2}-a^{2}} \right)+\gamma_{2}2\pi\left( r_{2}^{2}+r_{2}\sqrt{r_{2}^{2}-a^{2}} \right)+\gamma_{b}2\pi\left( r_{b}^{2}-r_{b}\sqrt{r_{b}^{2}-a^{2}} \right)$ | (S.9) |
| --- | --- |

which is similarly implemented by Villar *et al* for aqueous DIBs in oil.(1) The equilibrium model consists of minimizing the function$f$ by constraining the droplet volumes, $V_{1}$ and $V_{2}$, by equations

| $V_{1}=\frac{4}{3}\pi r_{1}^{3}+\frac{1}{3}\pi\left[ \left( 2r_{b}^{2}-2r_{b}\sqrt{r_{b}^{2}-a^{2}}-a^{2} \right)\left( 2r_{b}+\sqrt{r_{b}^{2}-a^{2}} \right)-\left( 2r_{1}^{2}-2r_{1}\sqrt{r_{1}^{2}-a^{2}}-a^{2} \right)\left( 2r_{1}+\sqrt{r_{1}^{2}-a^{2}} \right) \right]$ | (S.10) |
| --- | --- |
| $V_{2}=\frac{4}{3}\pi r_{2}^{3}-\frac{1}{3}\pi\left[ \left( {2r}_{2}^{3}-2r_{2}\sqrt{r_{2}^{2}-a^{2}}-a^{2} \right)\left( 2r_{2}+\sqrt{r_{2}^{2}-a^{2}} \right)+\left( 2r_{b}^{2}-2r_{b}\sqrt{r_{b}^{2}-a^{2}}-a^{2} \right)\left( 2r_{b}+\sqrt{r_{b}^{2}-a^{2}} \right) \right].$ | (S.11) |

This model is only valid under specific conditions however, which include: (1) the effect of droplets wetting to the surface is neglected, (2) on the given timescale there is no work done on the droplets by water permeating the membrane due to osmotic stress or Laplace pressure gradients, (3) the temperature is held constant, (4) the droplets are small enough to neglect the effect of gravity, (5) there is no substantial droplet evaporation, and (6) there is no electrochemical potential. The solution to this minimization problem can be implemented in MATLAB using the “*fmincon*” function employing the *interior point* *method*.

This can also be partially solved analytically by the method of Lagrange Multipliers with multiple constraints. The system of equations is given by the equation

| $\frac{\partial f}{\partial x}-\lambda\frac{\partial V_{1}}{\partial x}-\mu\frac{\partial V_{2}}{\partial x}=0$ | (S.12) |
| --- | --- |

where$x\in\left\{ r_{1},r_{2},r_{b},a \right\}$. The solution yields the values of the Lagrange Multipliers,

| $\lambda=-2\frac{\gamma_{1}}{r_{1}}$ | (S.13) |
| --- | --- |
| $\mu=-2\frac{\gamma_{2}}{r_{2}}$ | (S.14) |

which happen to be the Laplace pressure of the droplets. This leads to two non-linear systems of equations,

| $\frac{\gamma_{b}r_{1}r_{2}}{r_{b}}-\gamma_{1}r_{2}+\gamma_{2}r_{1}=0$ | (S.15) |
| --- | --- |

| $-\gamma_{1}\left( \frac{r_{1}}{\sqrt{r_{1}^{2}-a^{2}}} \right)+\gamma_{b}\left( \frac{r_{b}}{\sqrt{r_{b}^{2}-a^{2}}} \right)-\gamma_{2}\left( \frac{r_{2}}{\sqrt{r_{2}^{2}-a^{2}}} \right)-\frac{\gamma_{1}}{r_{1}}a^{2}\left[ \frac{1}{\sqrt{r_{b}^{2}-a^{2}}}-\frac{1}{\sqrt{r_{1}^{2}-a^{2}}} \right]+\frac{\gamma_{2}}{r_{2}}a^{2}\left[ \frac{1}{\sqrt{r_{2}^{2}-a^{2}}}+\frac{1}{\sqrt{r_{b}^{2}-a^{2}}} \right]=0$ | (S.16) |
| --- | --- |

that must be solved numerically. The correctness of the system of equations can be verified rather simply. For the case when the droplets are the same size with symmetric surface energies, the equation

| $\gamma_{b}=\frac{2\gamma_{1}}{r_{1}}\frac{\left( r_{1}^{2}-a^{2} \right)}{\sqrt{r_{1}^{2}-a^{2}}}=2\gamma_{1}\frac{\sqrt{r_{1}^{2}-a^{2}}}{r_{1}}=2\gamma_{1}\cos\theta$ | (S.17) |
| --- | --- |

becomes the well-known Young’s equation (3.1). One useful relation that can be extracted from this solution is the case when $a$ is zero, *i.e.* no DIB is formed. The equation

| $\gamma_{b}\geq\gamma_{1}+\gamma_{2}$ | (S.18) |
| --- | --- |

implies that when the bilayer surface energy is larger than the sum of the individual monolayers surface energies that a DIB is not energetically favourable. For bilayer surface energies that are increasingly smaller than the sum of the monolayer energies, the bilayer interface tends to ‘zip up’ so that the interfacial area increases to the limit that the two hemispheres form a single sphere where$r_{1,2} = a$ as shown in **Figure 3.2**. Alternatively, if the monolayer surface energies tend to zero, then the DIB will ‘unzip’ completely so that $a=0$.

**Free Energy Model Error Analysis**

An error analysis on bilayer surface energy from equation (3.1) and (3.2) is given for the equation

| $\gamma_{b}=\gamma_{2}\left( \frac{\cot\Theta_{1}\sin\Theta_{2}+\cos\Theta_{2}}{\cos\Theta_{b}-\cot\Theta_{1}\sin\Theta_{b}} \right)$ | (S.19) |
| --- | --- |

As a function of droplet 2 surface energy$\gamma_{2}$. The error on $\gamma_{b}$ is found with a standard error propagation. The partial derivatives with respect to$\Theta_{1}$,

| $\frac{\partial\gamma_{b}}{\partial\Theta_{1}}=-\gamma_{2}\left( \csc^{2} \left( \Theta_{1}-\Theta_{b} \right)\sin\left( \Theta_{2}+\Theta_{b} \right) \right)$ | (S.20) |
| --- | --- |

with respect to$\Theta_{2}$,

| $\frac{\partial\gamma_{b}}{\partial\Theta_{2}}=\gamma_{2}\left( \cos\left( \Theta_{1}+\Theta_{2} \right)\csc\left( \Theta_{1}-\Theta_{b} \right) \right)$ | (S.21) |
| --- | --- |

and with respect to $\Theta_{b}$,

| $\frac{\partial\gamma_{b}}{\partial\Theta_{b}}=\gamma_{2}\left( \sin\left( \Theta_{1}+\Theta_{2} \right)\cot\left( \Theta_{1}-\Theta_{b} \right)\csc\left( \Theta_{1}-\Theta_{b} \right) \right)$ | (S.22) |
| --- | --- |

are applied to the simplified error propagation equation

| $s_{\gamma_{b}}=\sqrt{\left( \frac{\partial\gamma_{b}}{\partial\gamma_{2}}s_{\gamma_{2}} \right)^{2}+\left( \frac{\partial\gamma_{b}}{\partial\Theta_{1}}s_{\Theta_{1}} \right)^{2}+\left( \frac{\partial\gamma_{b}}{\partial\Theta_{2}}s_{\Theta_{2}} \right)^{2}+\left( \frac{\partial\gamma_{b}}{\partial\Theta_{b}}s_{\Theta_{b}} \right)^{2}}.$ | (S.23) |
| --- | --- |

Based on the image resolution, the spatial dimensions are accurate to ±2 $\mu m$, which can be applied to equation (3.4), where we have the error on the angle as,

| $s_{\Theta_{1,2,b}}=\sqrt{\left( \frac{s_{a}}{r_{1,2,b}\sqrt{1-\left( \frac{a}{r_{1,2,b}} \right)^{2}}} \right)^{2}+\left( \frac{s_{r_{1,2,b}}a}{\left( r_{1,2,b} \right)^{2}\sqrt{1-\left( \frac{a}{r_{1,2,b}} \right)^{2}}} \right)^{2}}.$ | (S.24) |
| --- | --- |

However, the overall error is mainly driven by the error on $\gamma_{2}$, where the literature value of $\gamma_{2}=1.18 mN m^{-1}$ with an error of $0.067 mN m^{-1}$.(2)

1. Villar G, Heron AJ, Bayley H. Formation of droplet networks that function in aqueous environments. Nat Nano. 2011;6(12):803-8.

2. Taylor GJ, Venkatesan GA, Collier CP, Sarles SA. Direct in situ measurement of specific capacitance, monolayer tension, and bilayer tension in a droplet interface bilayer. Soft Matter. 2015;11(38):7592-605.
